# Gene Gun Bombardment with DNA-Coated Golden Particles Enhanced the Protective Effect of a DNA Vaccine Based on Thioredoxin Glutathione Reductase of *Schistosoma japonicum*

**DOI:** 10.1155/2013/952416

**Published:** 2012-12-31

**Authors:** Yan Cao, Bin Zhao, Yanhui Han, Juan Zhang, Xuezhen Li, Chunhui Qiu, Xiujuan Wu, Yang Hong, Dezhou Ai, Jiaojiao Lin, Zhiqiang Fu

**Affiliations:** Shanghai Veterinary Research Institute, Chinese Academy of Agricultural Sciences, Key Laboratory of Animal Parasitology of Ministry of Agriculture, 518 Ziyue Road, Minhang, Shanghai 200241, China

## Abstract

Schistosomiasis, caused by infection with *Schistosoma* species, remains an important parasitic zoonosis. Thioredoxin glutathione reductase of *Schistosoma japonicum *(SjTGR) plays an important role in the development of the parasite and for its survival. Here we present a recombinant plasmid DNA vaccine, pVAX1/SjTGR, to estimate its protection against *S. japonicum* in BALB/c mice. The DNA vaccine administrated by particle bombardment induced higher protection than by intramuscular injection. All animals vaccinated with pVAX1/SjTGR developed significant specific anti-SjTGR antibodies than control groups. Moreover, animals immunized by gene gun exhibited a splenocyte proliferative response, with an increase in IFN-**γ** and IL-4. The recombinant plasmid administrated by gene gun achieved a medium protective efficacy of 27.83–38.83% (*P* < 0.01) of worm reduction and 40.38–44.51% (*P* < 0.01) of liver egg count reduction. It suggests that different modes of administering a DNA vaccine can influence the protective efficacy induced by the vaccine. Interestingly, from the enzymatic activity results, we found that worms obtained from pVAX1/SjTGR-vaccinated animals expressed lower enzymatic activity than the control group and the antibodies weakened the enzymatic activity of SjTGR *in vitro*, too. It implies that the high-level antibodies may contribute to the protective effects.

## 1. Introduction

Schistosomiasis is an important disease distributed in many parts of the world, most of which are the places with poor sanitation or irrigation areas, and it is estimated that 779 million people are at risk of schistosomiasis [1]. *S. japonicum* is the most difficult form of schistosomiasis to control among the 5 *Schistosoma* species which infect humans [2–4]. Schistosomiasis is a chronic and debilitating disease [5, 6] which is always accompanied by emaciation and anemia, and even death. 

Over the past decades of years, the Chinese government has implemented several control programs, including community-based praziquantel chemotherapy [7], health education, improved sanitation, environmental modification, and snail control. However, schistosomiasis remains an important public health concern in China. As snail control [8] is always difficult to achieve, and praziquantel has no effect on reinfection [9, 10], the disease is difficult to control. Therefore, a complementary approach to integrate chemotherapy, vaccination for example, is needed.

Since the 20th century, scientists have been trying to develop an effect vaccine against *S. japonicum* for field use [11–13], mainly for yellow cattle and water buffalos. Through decades of efforts, several kinds of vaccines have been developed, including cercariae-attenuated vaccines [14], natural or recombinant protein vaccines, nucleic acid vaccines, and multivalent affiliate vaccines. Currently, DNA vaccines have received increased attention and are considered advantageous compared to other vaccine preparations [15, 16], for low cost and easy preparation. However, the mode of delivering a DNA vaccine can influence the effect induced by the vaccine [17].

The schistosome tegument is a single syncytium that covers the surface of the parasite body [18]. Although there remains many unresolved questions in relation to the structure and function of the tegument, the dynamic host-interactive layer tegument is believed to involve in nutrient uptake, immune evasion and modulation, sensory reception, and signal transduction, and is important from a vaccine perspective [19–22]. A number of described vaccine candidates are membrane proteins [23, 24], muscle proteins [25], and enzymes [26–28]. Thioredoxin glutathione reductase of *S. japonicum* (SjTGR) is also a tegument antigen mainly distributed in the tegument of adult worms [29]. Adult schistosome worms, which reside in the hepatic portal system, are exposed to reactive oxygen compounds from metabolism and the host immune response. In eukaryocyte, two major systems, the thioredoxin (Trx) system and the glutathione (GSH) system, exist to detoxify reactive oxygen species (ROS). However, it has been proved that there are no separate Trx reductase and GSH reductase enzymes in *S. japonicum*, instead of the linked thioredoxin-glutathione system (TGR) [30]. As such, this union enzyme, thioredoxin glutathione reductase, is thought to be an attractive vaccine antigen candidate. 

In this paper, a recombinant DNA plasmid was constructed containing a complete open reading fragment of SjTGR and immunized with two different modes, particle bombardment, and needle inoculation to evaluate the ability to protect BALB/c mice against *S. japonicum* challenge and explore the conceivable immune protective mechanism. 

## 2. Material and Methods

### 2.1. Experimental Mice and Parasites

Male BALB/c mice, 6–8 weeks old, were purchased from Slac Animal Laboratory (Shanghai, China). The freshwater snail, *Oncomelania hupensis*, was maintained in the Shanghai Veterinary Research Institute, Chinese Academy of Agricultural Sciences. Cercariae were collected by exposing infected snails to light and the number and viability of cercariae were determined under a light microscope before challenge. Animal care and all procedures involving animals were conducted according to the principles of Shanghai Veterinary Research Institute for the Care and Use of Laboratory Animals.

Specific anti-SjTGR serum was collected from BALB/c mice thrice immunized with recombinant protein SjTGR.

### 2.2. Construction of Recombinant Plasmid DNA

The eukaryotic expression plasmid, pVAX1, which contains the strong cytomegalovirus (CMV) promoter and bovine growth hormone (BGH) polyadenylation signal, was used as the vector. The entire SjTGR open reading fragment was amplified from the *S. japonicum* adult worm cDNA library, with primers: 5′-CGC*GGATCC*ATGCCTCCGATTGAT-3′ and 5′-GC*CTCGAG*TCAGCAACCGGTTACC-3′ and subcloned into cleaved pVAX1 to construct the recombinant expression plasmid, pVAX1/SjTGR.

The recombinant plasmid was sequenced to ensure the insert sequence was cloned correctly. Then, the expression plasmid was transferred into DH5*α*, a type of competent cell, for large-scale preparation and purification using a Qiagen Plasmid Maxi Kit (Qiagen), followed by the manufacturer's protocol. 

### 2.3. Expression in 293T Cells

Transfections of plasmid (pVAX1/SjTGR, pVAX1) were done by Lipofectamine 2000 (Invitrogen) according to the manufacturer's protocol to detect gene expression in 293T cells. One day before transfection, cells were plated into a 6-well plate in 2 mL/well of growth medium without antibiotics so that the cells will be 90%–95% confluent when transfection was performed. For each well, 5 *μ*L Lipofectamine 2000 and 10 *μ*g DNA were mixed gently and incubated for 20 min at room temperature. Then, the complex was volume to 2 mL with Opti-MEM and added to the monolayer of cells in each well. 

After incubating cells at 37°C in a 5% CO_2_ incubator for 48 h, the monolayer cells were fixed with 80% ethanol, and thrice washed with PBS-0.05%/Tween-20 (PBST). Specific anti-SjTGR serum was added to each well and incubated at 37°C for 2 h, then thrice washed with PBST. After that, Cy3-labeled goat anti-mouse antibodies (Beyotime) were used as secondary antibodies at a dilution of 1 : 5000 and added to each well. After 1 h incubation at 37°C keeping in dark, the plates were thrice washed again. Finally, the potential protein in the cells was detected using a converted fluorescence microscope. The tests were assayed in triplicate.

### 2.4. Immunization with the Helios Gene Gun System

Cartridges were prepared prior to the day of the experiments, followed by general methods [31]. First, the amount of DNA and gold required for each transformation was calculated. The DNA loading ratio (DLR) used was 5 *μ*g DNA/mg gold, and the microcarrier loading quantity (MLQ) was 0.5 mg/cartridge. For the duration of producing bullets, polyvinylpyrrolidone (PVP) (Sigma) served as an adhesive. At higher discharge pressures from the nitrogen source, DNA mixed with nanolevel gold particle was coated in the walls of the tubes (BioRad) and is referred to as bullet. Similarly, at higher discharge pressures from the helium source, murine abdominal epidermis was bombarded with the Helios gene gun system (BioRad). The optimum pressure for mice was determined to occur at 600 psi. 

### 2.5. Immunization Schedule and Challenge Infection

**Figure 1:**
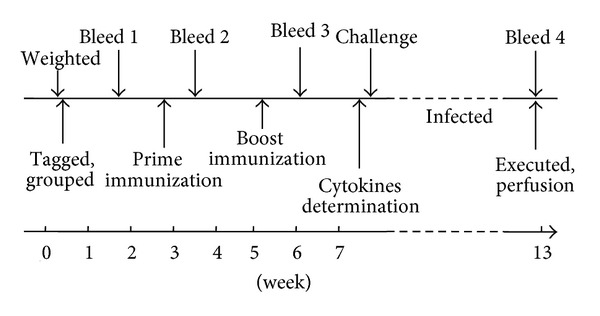
Immunization schedule and challenge infection of animals.

Two mice vaccinations were carried out in this study. In trail 1, fifty-male BALB/c mice were randomly divided into 5 groups (10 each group, pVAX1/i.m., pVAX1/SjTGR/i.m., pVAX1/g.g., pVAX1/SjTGR/g.g., PBS). All mice were given 2 intramuscular immunizations 3 weeks apart called prime-boost inoculation by two different modes, particle bombardment and needle inoculation. In trail 2, forty-five male BALB/c mice were randomly divided into 3 groups (15 each group, pVAX1/g.g., pVAX1/SjTGR/g.g., and PBS) and immunized with a gene gun with the same schedule. Ten days after each immunization in the trail 2, blood from each animal was collected. Serums were separated and stored at −20°C for antibody assays and cytokine detections. Two weeks later, mice in each group of the two trails were infected with 40 ± 2  *S. japonicum* cercaria and sacrificed 6 weeks after challenge and blood was collected. The total worm and liver egg burden was determined (Figure 1).

### 2.6. Detection of Specific Antibodies in Serum by Enzyme-Linked Immunosorbent Assay (ELISA)

In trail 2, the levels of specific IgG antibodies against SjTGR were detected by ELISA following standard methods [32]. A 96-well flat-bottomed plate was coated with recombinant protein SjTGR at 4°C overnight (1 *μ*g/well), thrice washed with PBS-0.05%/Tween-20 (PBST), blocked with 150 *μ*L/well of PBST-1.5% (m/v) normal bovine serum albumin (BSA) for 2 h at room temperature (25°C), then thrice washed with PBST. The serum samples collected in the previous section were diluted with PBST in 1 : 100, added to the plate (100 *μ*L/well), incubated at 37°C for 2 h, and thrice washed again with PBST. Horseradish peroxidase labeled goat anti-mouse IgG, IgG1, and IgG2a (BD Pharmingen) were used as secondary antibodies at a dilution of 1 : 5000 and added at 100 *μ*L/well. After a 1 h incubation at 37°C, the plates were thrice washed and the substrate, 3,3′5,5′-tetramethyl benzidine dihydrochloride (TMB), was added (100 *μ*L/well). The plates were incubated for 10 min at room temperature in the dark and the reaction was stopped using 2 M H_2_SO_4_ (50 *μ*L/well). All of the samples were assayed in triplicate. The results were detected in a microplate reader (BioTek) and the absorbance was measured at 450 nm. 

### 2.7. Calculation of the Percentage of CD4+ and CD8+ Cells and Cytokine Determination by Flow Cytometry

Five mice in each group in trail 2 were sacrificed 2 weeks after the booster immunization and splenocytes were collected. 1 mL RPMI 1640 medium (Gbico) with 10% FBS was added to each spleen and grinded. After grinding, 1.5 mL FACS lysing solution (BD Pharmingen) was added to the cells, and thrice washed by centrifuging at 3000 g for 5 min at 4°C. The cells were adjusted to 10^7^/mL and cultured overnight and the next day stimulated with 2.5 *μ*L (1 *μ*L/mL) PMA (Sigma) 2 *μ*L (1 *μ*g/mL) Ionomycin (Sigma) and 3.4 *μ*L Monensin (eBioscience), for 6 h at 37°C in a 5%  CO_2_ incubator. Then, 0.25 *μ*g PE-labeled Cy5 CD3 *ε* and 0.25 *μ*g PE-labeled Cy7 CD8 *α* (BD Pharmingen) were added to each sample, and incubated at 25°C for 20 min at dark, thrice washed as usual. Then 1 mL dyeing buffer was used to wash the cells for three times as usual. After that, 0.5 mL cell-fixed liquid was added to each sample for 20 min and washed thrice as usual. Cells were resuspended with 100 *μ*L permeabilization for another 20 min. Then for each sample, 0.25 *μ*g PE-labeled anti-IL-4 antibodies and 0.25 *μ*g FITC-labeled anti-IFN-*γ* antibodies (BioLegend) were added and thrice washed as usual. Finally, 0.6 mL cell staining buffer was added to resuspend the cells, and a flow cytometry system (Beckman) was used to detect the interferon-gamma (IFN-*γ*) and interleukin (IL)-4 levels. The criteria for this study were set according to the blank measurement. The ratio of CD4+ and CD8+ T cells in total cells was examined and the rates of T cells producing IFN-*γ* or IL-4 were reported.

### 2.8. Count of Worm and Liver Egg Burden

Forty-two days after challenge, all mice (10 in each group) were euthanized in the two independent trails, and worms were collected by perfusion from the hepatic portal system then counted.

To determine the liver egg burden, each mouse liver were weighed, homogenized, and digested for approximately 1 h at 56°C with 10 mL 10% NaOH. The suspensions were agitated, and 1-mL aliquots, collected from the middle of each tube, were transferred into 1.5-mL Eppendorf tubes and spun at a low speed to sediment the particles. Pellets were then resuspended in 200 *μ*L of PBS and the egg counts were determined under a microscopy.

Reductions in the parasite burden were calculated as follows: worm reduction rate (%) = ((average number of recovered worms of control group-average number of recovered worms of experimental group)/average number of recovered worms of control group) × 100; and egg reduction rate (%) = ((average number of eggs/g liver in control group-average number of eggs/g liver in experimental group)/average number of eggs/g liver of control group) × 100.

### 2.9. Enzyme Activity Analysis by Thioredoxin Reductase Assay Kit

The enzyme activity analysis was referenced to Han [29]. Six-week-old worms were collected in trail 2 and stored at −80°C with Dulbecco's phosphate buffered saline and protease inhibitor cocktail (Sigma) at a ratio of 1 : 1000. Then, all of the worms (50 worms in each group) were grinded with Ready Prep Mini Grinders (BioRad) on ice and all the samples were cracked thoroughly by freezing and thawing three times. The thawed lysates were centrifuged at 10000 ×g for 20 min at 4°C and the supernatants containing thioredoxin glutathione reductase were used to detect the enzymatic activity in each individual group.

The enzyme activity was assessed using a Thioredoxin Reductase Assay Kit (Sigma) for an easy and simple colorimetric assay [33]. It is based on the reduction of 5,5′-dithiobis(2-nitrobenzoic) acid (DTNB) with NADPH to 5-thio-2-nitrobenzoic acid (TNB), which produces a strong yellow color that is measured at 412 nm. Components were added to a cuvette with a final volume of 1 mL, and 30 *μ*L of DTNB in DMSO (100 mM) was added immediately before detection with a Thermo NanoDrop ND-2000C (Thermo). The enzymatic kinetic program was set as follows using the spectrophotometer: delay = 120 sec, interval = 10 sec, and number of readings = 6. During the test, an inhibitor solution for specific inhibition of mammalian thioredoxin reductase contained in the kit was used, to determine the reduction of DTNB due only to thioredoxin reductase activity of SjTGR. All of the samples were detected in triplicate independently. And the data was calculated as the following computational formula:

(1) $$\begin{array}{c} \text{Unit}/\text{mL}=\frac{\Delta {\text{A}}_{412}/\min\left(\text{thioredoxin}\;\text{reductase}\right)\times \text{dil}\times \text{vol}}{\text{enzol}}, \end{array}$$

where, ΔA_412_/min (thioredoxine reductase) = [ΔA_412_/min (sample) − ΔA_412_/min (sample + inhibitors)], dil = sample dilution factor, vol = volume of reaction in mL, and enzol = volume of enzyme in mL.

An *in vitro* test was carried out to evaluate the weakened effect of specific anti-SjTGR serum to thioredoxin reductase. Forty-two-day-old worms were carefully collected and soluble adult worm antigen preparations (SWAPs) were extracted using the above methods. Anti-SjTGR and normal mouse sera were added to SWAPs (1 mg/mL) at a ratio of 1 : 1, and both of the mixtures were incubated at room temperature for 2 h. The enzymatic activity of SjTGR was then detected with the methods described above.

### 2.10. Statistical Analysis by SPSS

All data were compared by analysis of variance (ANOVA) and Student's *t*-test using SPSS v.12.0 software. *P* values < 0.05 were considered statistically significant.

## 3. Results

### 3.1. Transient Expression of Recombinant Plasmid in 293T Cells

The SjTGR entire open reading frame amplified by PCR with special primers was subcloned into the plasmid pVAX1 with T4 DNA ligase and was confirmed correct by restriction enzyme digestion and sequencing. Then, the recombinant plasmids were transiently transfected into 293T cells.

**Figure 2:**
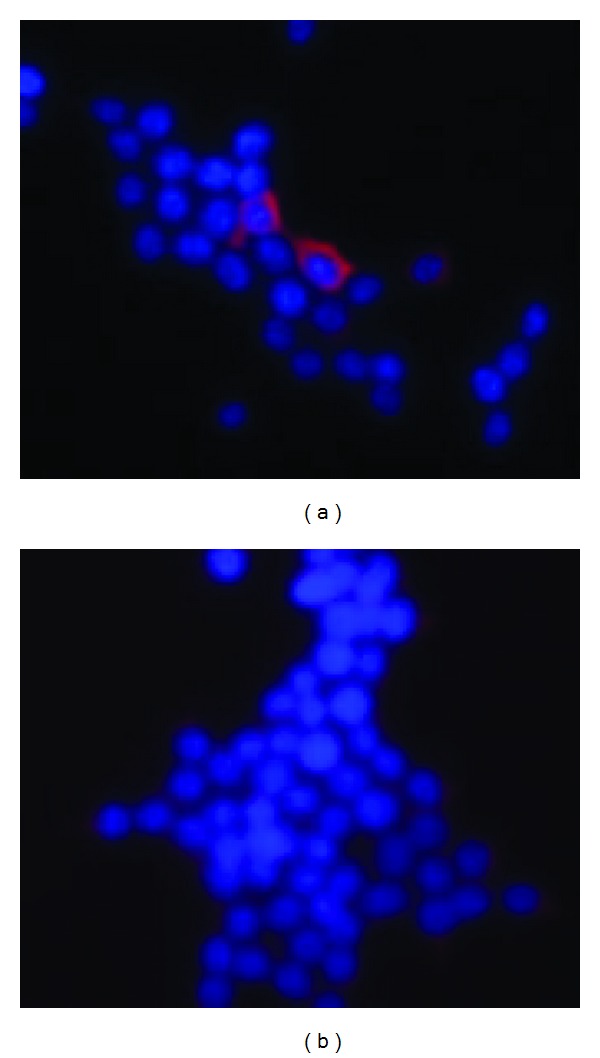
Protein expression of SjTGR in 293T cells. (a) Red fluorescence was observed on the 293T cells after pVAX1/SjTGR plasmid transfection; (b) negative control transfected with naked pVAX1 plasmid alone, and no fluorescence was observed.

Forty-eight hours after transfection of recombinant plasmid pVAX1/SjTGR, the cells were fixed to detect the expression of the protein of interest. Red fluorescence was observed on the cells transferred with pVAX1/SjTGR (Figure 2(a)), but not on those transferred with pVAX1 vector DNA alone (Figure 2(b)). 

### 3.2. Evaluation of Protective Efficacy Induced by pVAX1/SjTGR

The total worm burden and eggs per gram (EPG) in each group, as well as the percent reduction in the worm burden and EPG in the vaccinated group compared with PBS control group are summarized in Table 1. In trail 1, animals administrated with particle bombardment induced better protective efficacy. Mice vaccinated with pVAX1/SjTGR by bombarding murine epidermis with the Helios gene gun system resulted in a significant worm burden reduction of 27.83% (*P* < 0.01) and 38.83% (*P* < 0.01) compared to the PBS control group in two independent trails, respectively. And no protection was observed in pVAX1/SjTGR/i.m. group as well as in two pVAX1 vaccinated groups. Mice in the vaccinated group by gene gun resulted in a significant liver egg burden reduction of 40.38% (*P* < 0.01) and 44.51% (*P* < 0.01) to the blank control group, in trail 1 and 2, respectively. Significant egg reduction also observed in pVAX1/SjTGR/i.m. group, but not in pVAX1 vaccinated groups.

### 3.3. Antibody Assay

**Figure 3:**
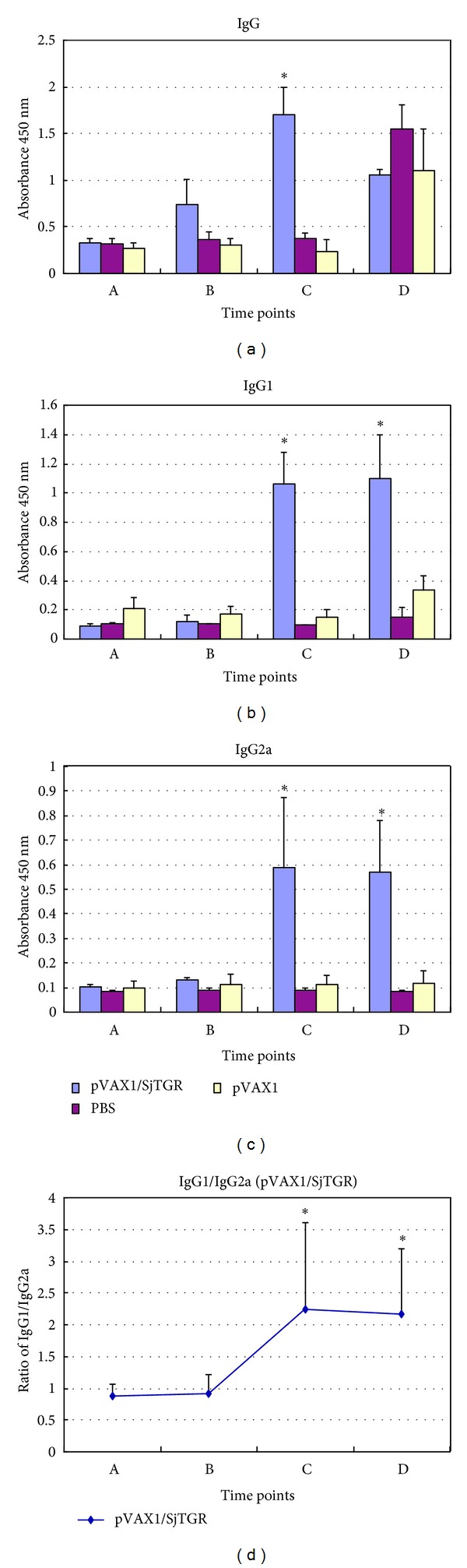
Levels of antibodies of mice immunized with gene gun in each group by ELISA. Figures (a), (b), and (c) display detection of specific IgG, IgG1, and IgG2a antibodies, respectively. Figure (d) displays the ratio of IgG1-to-IgG2a in pVAX1/SjTGR group. A, B, and C indicated before vaccination, 10 d after prime vaccination, and 10 d after boost vaccination. D indicates that 42 d after challenged with cercariae. The results are presented as mean ± SD for each group (pVAX1/SjTGR, pVAX1, PBS). The asterisk (*) indicates significantly increased antibody levels of serum collected from pVAX1/SjTGR compared with both pVAX1 and PBS control.

Total IgG antibodies and its subtypes of IgG1 and IgG2a were detected by ELISA in trail 2, as described. SjTGR-specific IgG antibody was detected 10 days after immunization, significantly increased after boost vaccination, in the pVAX1/SjTGR immunized mice, and the specific antibody had no obvious change in mice that received pVAX1 vector DNA or PBS only (Figure 3(a)). 

**Figure 4:**
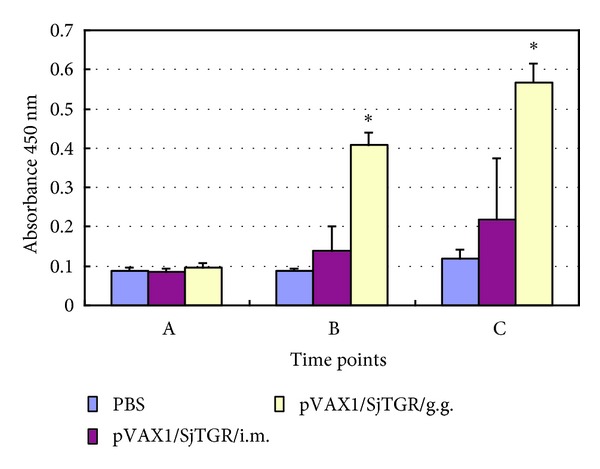
Levels of antibodies of mice immunized with two different modes by ELISA. A, B and C indicated that before vaccination, 10d after prime vaccination and 10d after boost vaccination. The asterisk (*) indicates significantly increased antibody levels compared with PBS control.

Both SjTGR-specific IgG1 (Figure 3(b)) and IgG2a antibodies (Figure 3(c)) increased significantly after the booster immunization with pVAX1/SjTGR, and the level of IgG1 was higher than IgG2a; the IgG1/IgG2a ratio was significantly increased after boost immunization (Figure 3(d)). No significant changes were noted in the two control groups in specific IgG1 and IgG2a antibody levels. We also found that different inoculation modes can induce different levels of antibodies, and the level of specific IgG antibody induced by recombinant plasmids pVAX1/SjTGR is significantly higher when delivered by gene gun than that by i.m. (Figure 4).

### 3.4. T Cell Subsets and Cytokine Determination

**Figure 5:**
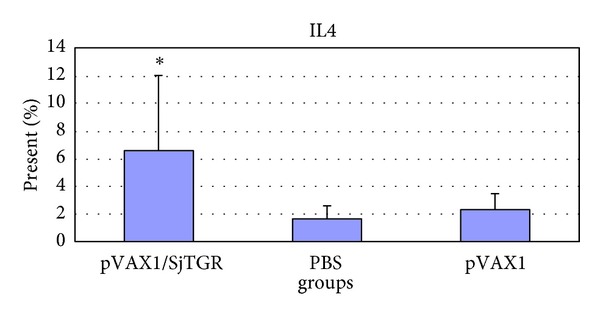
The level of IL-4 in each group. The asterisk (*) indicates the significant increase of the level of IL-4 in pVAX1/SjTGR-immunized group compared with PBS and pVAX1 group (*P* < 0.05).

**Figure 6:**
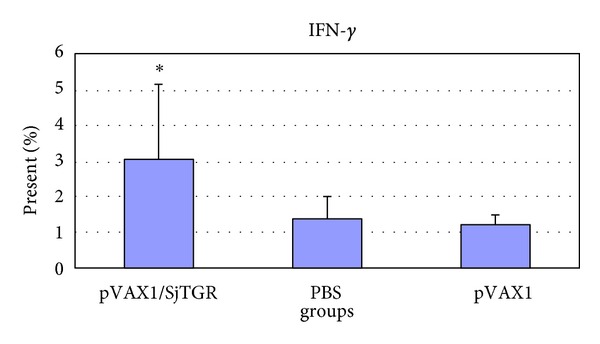
The level of IFN-*γ* in each group. The asterisk (*) indicates the significant increase of the level of IFN-*γ* in pVAX1/SjTGR-immunized group compared with PBS and pVAX1 group (*P* < 0.05).

After the last immunization, the splenic lymphocytes of animals from each group in trail 2 were collected. Grinded cells were cultured, stimulated, stained by fluorescent-labeled antibodies, and detected with a flow cytometry system (FMC). In this paper, particle bombardment immunization pushs T cells forward to CD4+ T cells (Table 2). And the percent of cells producing IL-4 (Figure 5) or IFN-*γ* (Figure 6) in the pVAX1/SjTGR-vaccinated group were significantly increased compared to those in pVAX1 or PBS group.

### 3.5. Activation Changes of Enzyme

**Figure 7:**
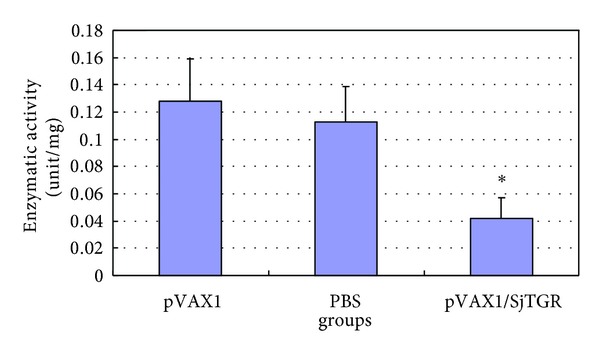
Enzymatic activity detection of thioredoxin reductase of SjTGR in each immunization group. The results are presented as mean ± SD after three independent tests. The asterisk (*) indicates the significant decrease of the enzymatic activity of SjTGR in pVAX1/SjTGR-immunized group compared with PBS and pVAX1 group (*P* < 0.05).

SWAPs were extracted from worms in each individual group and enzymatic activity was detected. The detectable products of substrate were increased with incubation time. With same amount of SWAPs, the thioredoxin reductase (TR) enzymatic activity in the worms from pVAX1/SjTGR-immunized group expressed much lower enzymatic activity than that of the pVAX1 or PBS control groups (Figure 7).

**Figure 8:**
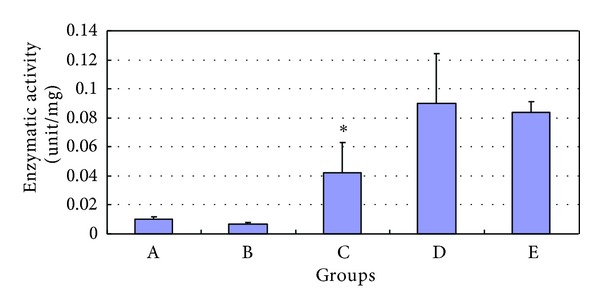
The TR enzymatic activity of SWAP after serum-treatment. The results are presented as mean ± SD after three independent tests. A, B, C, D, and E indicated that anti-SjTGR serum, normal mouse serum, anti-SjTGR serum-treated SWAP, normal mouse serum-treated SWAP, and untreated SWAP. The asterisk (*) indicates the significant decrease of the enzymatic activity of SWAP after serum treatment compared with normal SWAP and SWAP treated with normal serum (*P* < 0.05).

Furthermore, SWAPs treated with anti-SjTGR antibodies expressed a lower TR enzymatic activity than untreated SWAPs, and the enzyme activity was not affected after incubating with normal mouse serum, indicating that anti-SjTGR antibodies influenced the thioredoxin reductase activity of SjTGR to catalyze DNTB into NTB, which can be measured at 412 nm (Figure 8).

## 4. Discussion

Thioredoxin glutathione reductase of *Schistosome japonicum *is a membrane protein of about 65 kDa, which is considered as a promising vaccine candidate antigen based on its immunogenicity [29] and its important role in parasite metabolism as a vital enzyme to balance redox equilibrium [34]. In the current study, we focused on evaluation of a recombinant plasmid pVAX1/SjTGR as a DNA vaccine based on SjTGR against schistosomiasis japonicum with two different modes. And pVAX1 was chosen as the vaccination regimen because it is mentioned by FDA before (http://www.fda.gov/ohrms/dockets/ac/00/transcripts/3664t1_a.pdf). 

The main methods of delivery plasmid DNA into animals include intramuscular injection and intradermal delivery into skin by a gene gun system [35]. And the administration mode of delivering a DNA vaccine can influence the type of immune response [17] and somehow influence the result of immunoprotective efficacy. In this study, mice vaccinated with pVAX1/SjTGR by bombarding murine epidermis with the Helios gene gun system (BioRad) elicited a much stronger IgG antibody response specific for SjTGR by ELISA (Figure 3(a)), and achieved a prominent reduction of worm (27.83%, 38.83%, *P* < 0.01, Table 1) and liver EPG (40.38%, 44.51%, *P* < 0.01, Table 1), which was considered as a high-efficiency method for less cost (2.5 *μ*g/mouse), compared with needle injection (20 *μ*g/mouse). Yoshida et al. compared the two methods in reproducible induction of specific immune responses and found that gene gun DNA delivery appeared to bring about highly reproducible and reliable results while the results obtained by intramuscular inoculation vary significantly [36]. They also thought that intramuscular injection appears to favor Th1 responses, while gene gun prefers to promote Th2 responses. And our results are like theirs to some extent.

The DNA vaccine transferred into cells by gene gun bombardment with golden particles under a special-high pressure (600 psi) will be assimilated by professional antigen-presenting cells or some other cells, which can induce humoral and cellular immunity [37]. DNA-coated golden particles delivery by gene gun predominantly produces IgG1 and induces Th2-type responses [17]. In our experiment, the dose of DNA plasmid that used in particle bombardment is 8-fold less than needle injection. But the former received much better protection. And it is considered that the CpG motif provided by the vector pVAX1 can influence the immune response, too [38].

In this paper, mice immunized pVAX1/SjTGR by particle delivery induced both anti-IgG1 and IgG2a antibodies increasing. The antibodies induced by SjTGR DNA constructs were dominantly IgG1 type which is proved to be efficient in complement fixation and with cytophilic property in antibody-dependent cell-mediated cytotoxicity [39], and the result is the same with Da'dara et al. [40], who stated that gene gun immunization resulted in significantly higher levels of IgG1. And from the FMC results, we found that the ratio of CD4+ T cells/CD8+ T cells was promoted after boost immunization by gene gun, from the normal ratio (2 : 1) to about 2.7 : 1 (Table 2). The cell surface antigen CD4 is the receptor of major histocompatibility complex-II (MHCII), which is located on the surface of antigen presenting cells (APCs), such as dendritic cells (DCs). The activated CD4+ T cells work as three different subsets depending on their different functions. One of them is helper T cell (Th), which induced humoral and cellular immune responses by means of two kinds of Th cells. The Th1 cells mediate cytokine producing with the signal of IFN-*γ* level increasing, known as Th1 response, while the Th2 cells secrete IL-4 and other cytokines, named Th2 response [37, 41]. On the other hand, Th cells can activate B cells and regulate their differentiation and antibody producing. IL-4 can activate B lymphocytes to produce IgG1 subtype antibodies, which is beneficial to transform between antibody subtypes and enhances Th2-type immune responses [41, 42]. And mice immunized with the recombinant plasmid by gene gun elicited both Th1-type cytokine IFN-*γ* and Th2-type cytokine IL-4 secreting augment, suggesting that mice-inoculated pVAX1/SjTGR plasmid induced mixed Th1/Th2 immune responses. Sawant [43] suggested that IL-4 may be more appropriate as a genetic adjuvant than IFN-*γ* for ND (Newcastle) DNA vaccine. One strategy to improve DNA vaccine-induced immune responses is the utility of cytokine cDNA as a molecular adjuvant [44]. Coimmunization of these cytokine molecular cassettes is an effective method to modulate the direction of the immune responses (humoral or cellular immune response).

In addition, SWAPs were prepared to determine SjTGR thioredoxin reductase activity *in vitro*. SWAPs of worms from immunized animals expressed a much lower enzymatic activity in catalyzing DTNB into TNB (Figure  7) and SWAPs treated with specific anti-SjTGR serum displayed lower enzyme activity compared to untreated SWAP (Figure  8). This may be an important evidence to explain reduction of worms and eggs. We speculate that the complex of specific anti-SjTGR antibodies and the interest enzyme lead to the changes of spatial structure of SjTGR, which may be a good competitive inhibitor of interaction between the enzyme and substrate. Consequently, the development progress of worms may be blocked partially, which is responsible for the protective effect against schistosomiasis. On the other hand, the DNA vaccine inoculated by golden-particle bombardment induced much higher antibody level (Figure 4), which is probably beneficial to the protection.

In this study we investigated the protective ability of pVAX1/SjTGR as a DNA vaccine by inoculating with a gene gun system. We found that animals immunized with pVAX1/SjTGR induced both humoral and cellular immunity. And the enzymatic activity of SjTGR was weakened to some extent by high-titer antibodies. Based on our work, SjTGR may be considered as a prospective vaccine candidate antigen against schistosomiasis in a BALB/c mouse model.

## Figures and Tables

**Table 1:** Protective efficacy induced by pVAX1/SjTGR in mice.

Groups	Worm reduction		Egg reduction	
	Worm burden (mean ± SE)	Percent reduction in worm burden (%)^a^	Liver egg per gram (EPG) (mean ± SE)	Percent reduction in liver egg count (%)^b^
Trail 1				
pVAX1/SjTGR/g.g. (*n* = 10)^c^	15.6 ± 8.86*	27.83	31591.3 ± 14647.42*	40.38
pVAX1/g.g. (*n* = 10)	23.3 ± 6.85	−8.03	42995.7 ± 11730.94	18.86
pVAX1/SjTGR/i.m. (*n* = 10)	24.6 ± 12.18	−14.12	25558.7 ± 15171.82*	51.77
pVAX1/i.m. (*n* = 10)	23.7 ± 5	−8.09	57686.6 ± 20951.58	−8.86
PBS (*n* = 10)	21.6 ± 3.94	—	52989.6 ± 19448.42	—

Trail 2				
pVAX1/SjTGR/g.g. (*n* = 10)	12.6 ± 7.17*	38.83	33326.5 ± 6875.44*	44.51
pVAX1/g.g. (*n* = 10)	21.6 ± 8.67	−4.85	65934.8 ± 16702.02	−9.79
PBS (*n* = 10)	20.6 ± 4.90	—	60055.1 ± 19211.27	—

Differences were significant at *P* < 0.05 (*); data was presented in 95% confidence interval.

^a^Percent reduction was determined using total worms in immunized group compared to PBS group.

^b^Percent reduction was calculated using liver egg burden in immunized group compared to PBS group.

^c^The number of animals in each group when perfusion.

**Table 2:** T cell subsets after boost immunization by gene gun.

Groups	T cell subsets		Description (ratio)
	CD4+ T cells (%)	CD8+ T cells (%)
pVAX1/SjTGR	20.49	7.40	2.77 : 1
	19.04	7.26	2.54 : 1
	20.84	7.49	2.78 : 1

pVAX1	14.23	7.30	1.95 : 1
	16.19	7.54	2.15 : 1
	14.78	7.26	2.04 : 1

PBS	13.92	7.89	1.74 : 1
	16.42	8.72	1.88 : 1
